# Comparative analysis of PTH Washout and 99mTc-MIBI SPECT/CT in presurgical localization for patients with primary hyperparathyroidism

**DOI:** 10.1007/s40618-026-02873-9

**Published:** 2026-05-06

**Authors:** Maja Cieślewicz, Ewelina Szczepanek-Parulska, Agnieszka Witowska, Dorota Filipowicz, Ariadna Zybek-Kocik, Rafał Czepczyński, Marek Ruchała

**Affiliations:** 1 Department of Endocrinology, Metabolism and Internal Diseases, Doctoral School, Poznan University of Medical Sciences, Przybyszewskiego Street 49, 60-355 Poznań, Poland; 2 Department of Endocrinology, Metabolism and Internal Diseases, Poznan University of Medical Sciences, Poznań, Poland

**Keywords:** Parathyroid neoplasms, Parathyroid hormone, Hyperparathyroidism, Fine-needle aspiration, Technetium Tc 99m sestamibi

## Abstract

**Purpose:**

Our study aimed to evaluate the performance and concordance of parathyroid hormone wash-out (PTHw) and ^99m^Tc-MIBI+SPECT/CT (MIBI) in preoperative localization for patients with primary hyperparathyroidism (PHPT), and assess potential factors influencing their diagnostic utility including patient characteristics, lesion size and comorbidities.

**Methods:**

This was a real-life, retrospective study involving 125 participants, who underwent ultrasound-guided fine needle aspiration biopsy with PTHw determined by electrochemiluminescence (Elecsys analyser) and subtraction scintigraphy together with SPECT/CT images.

**Results:**

PTHw was positive in 84.8% and MIBI in 84.0% of cases, with concordant findings in 73.6% of patients. In MIBI-negative cases, PTHw accurately localized PA in 70.0%, while MIBI identified lesions in 68.4% of PTHw-negative cases. Larger lesions were associated with improved performance for both techniques, although a significant size-dependent effect (≥10 mm) was observed only for MIBI (χ^2^ = 3.92, p = 0.048; φ = -0.18). The right inferior gland was the most frequent lesion location detected by PTHw (28.0%). PTHw correlated positively with serum PTH (r=0.29), PTHw-to-serum PTH ratio (r=0.84), total calcium (r=0.27), ionized calcium (r=0.31) and alkaline phosphatase (r=0.31), and inversely with phosphate (r = -0.22) and vitamin D (r= -0.20).

**Conclusion:**

Our findings reinforce the complementary role of ultrasound-guided parathyroid hormone washout and ^99m^Tc-MIBI with SPECT/CT, in the preoperative PHPT imaging diagnostics, particularly when one modality yields negative or inconclusive results. Additionally, our results highlight the need for individualized imaging strategies based on specific patient profiles. Larger prospective studies with histopathological verification are warranted to corroborate these observations and refine preoperative localization protocols.

## Introduction

In the last decades, the incidence and prevalence of primary hyperparathyroidism (PHPT) have risen. This trend is likely attributable to the implementation of routine serum calcium evaluation, the expansion of osteoporosis screening and demographic shifts such as population aging and rising obesity rates [1, 2]. On the other hand, widespread neck ultrasound (US) availability leads to more common suspicion of parathyroid lesion responsible for PHPT (PL), as enlarged gland is strongly indicative of underlying pathology, what prompts further biochemical assessment [3–5].

The cause of PHPT is a benign single parathyroid adenoma in approximately 80% of cases and multiple adenomas or hyperplasia in 20%, while parathyroid carcinoma constitutes less than 1%, usually with a large lesion and severe hypercalcemia or hypercalcemic crisis [6].

PHPT is characterised by elevated or inappropriately normal parathormone (PTH) concentration, resulting in disturbances in calcium and phosphate metabolism [7, 8].

Following the biochemical PHPT diagnosis, surgery is the only curative treatment [9, 10]. The approach to parathyroidectomy varies regarding the ability to localize the affected gland preoperatively. For undetectable lesion location or multi-gland disease, bilateral exploration is mandatory, although it has a higher risk of complications, longer surgery duration, and extensive scarring. Accurate preoperative localization enables unilateral exploration or minimally invasive surgery, thereby reducing abovementioned risks [11, 12]. When two concordant imaging modalities identify the PL preoperatively, which is additionally confirmed by intraoperative PTH measurement, the cure rate is 90.3-100% [13–15]. This underscores the importance of accurate preoperative localization and highlights the need for ongoing optimisation of imaging techniques.

The standard for PL localization remains ^99m^Tc-MIBI scintigraphy. However, reported sensitivity varies widely (52.2–95%). When combined with single-photon emission computed tomography/computed tomography (SPECT/CT), this modality demonstrates higher diagnostic accuracy in identifying both typical and ectopic lesions [16–20].

Ultrasonography (US) is commonly used as the first-line imaging modality, which is indirectly attributable to its accessibility. The sensitivity of US for PL detection ranges from 64% to 93% [20, 21]. However, in conjunction with PTH measurement in washout fluid (PTHw) obtained via fine-needle aspiration biopsy (FNAB), the sensitivity increases to 97.6%, with a specificity approaching 100% [22–25]. FNAB is a widely used diagnostic procedure with a favourable safety profile and high diagnostic accuracy [26]. Although theoretical concerns regarding tumour seeding have been raised, available evidence indicates that this complication is exceedingly rare (approximately 0.003–0.009%) and does not appear to adversely affect patient outcomes [27–30].

Other promising imaging options include positron emission tomography with ^18^F-choline (^18^F-FCH PET/CT), with a sensitivity of 97% and specificity of 95%, or dynamic contrast-enhanced MRI (4D MRI), but neither is yet widely available [23]. Noteworthy, PET/CT with [^68^Ga] - prostate-specific membrane antigen (^68^Ga-PSMA) may be useful in PL detection, but more evidence is warranted [32].

Previous studies have shown that combining US with ^99m^Tc-MIBI scintigraphy enhances diagnostic effectiveness, yielding sensitivities and specificities of 90-100% [20, 33, 34]. The routine incorporation of SPECT/CT into planar scintigraphy protocols has further enhanced detection rates, highlighting the need for renewed evaluation of these modalities as complementary approaches in larger, rigorously defined patient cohorts.

The aim of this study was to assess the performance and concordance of PTHw and ^99m^Tc-MIBI+SPECT/CT (MIBI) in preoperative localization of PL. Additionally, we sought to identify potential factors influencing diagnostic utility of these methods including the lesion size, patient characteristics and comorbidities.

## Materials and methods

**Fig. 1:**
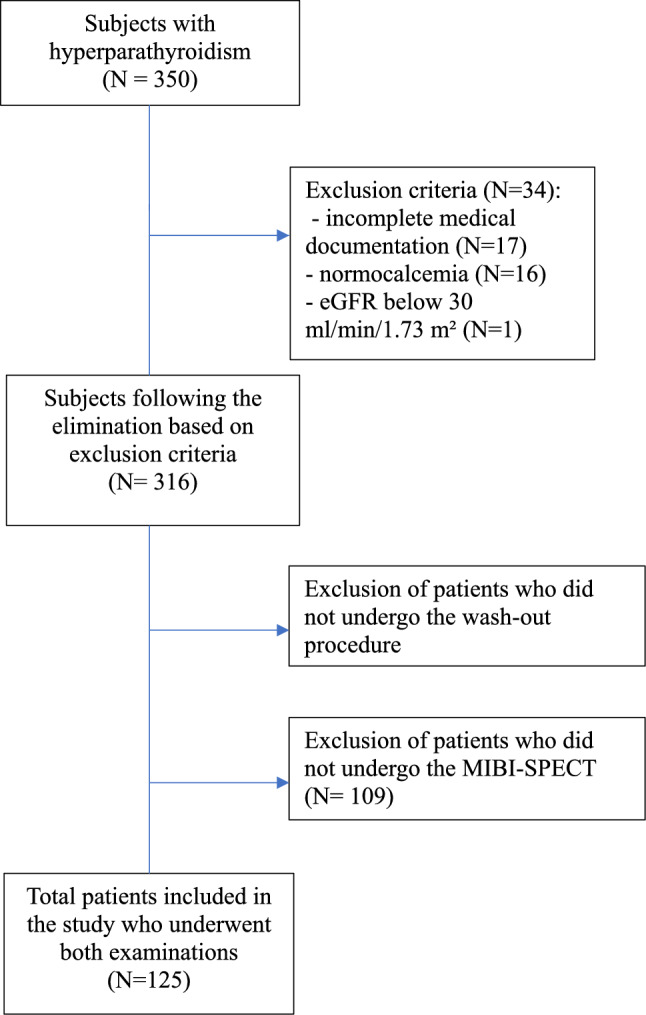
Flow diagram of patient enrolment and exclusion

We conducted a retrospective, real-life study involving 125 participants (110 women, 15 men). The median age was 59 years, ranging from 19 to 84. The analysis was based on data obtained from hospitalisation records at the Department of Endocrinology, Metabolism and Internal Medicine, as well as from the Outpatient Clinic of the Poznań University of Medical Sciences, covering the period from January 2019 to May 2025. All participants underwent a standard clinical protocol. We included patients with hypercalcaemia due to primary hyperparathyroidism, who had both ultrasound-guided parathyroid hormone washout (PTHw) and MIBI scintigraphy with SPECT/CT imaging performed. The study population consisted of 9 patients with genetically confirmed MEN1 mutations, 70 patients with MEN mutations excluded by genetic testing, and 46 patients who were not genetically evaluated due to the absence of clinical indications, in accordance with current recommendations [7, 8]. The exclusion criteria were: age under 18 years, any form of hyperparathyroidism other than primary, normocalcaemia, and an estimated glomerular filtration rate (eGFR) below 30 ml/min/1.73 m^2^. A flow diagram summarising patient selection was prepared to provide a detailed overview of the study cohort formation and is shown in Fig. 1.

All included patients underwent US-guided FNAB using the AIXPLORER system (Supersonic Imagine, Aix-en-Provence, France). A needle with an inner diameter of 0.5 mm was used to collect washout fluid, which was subsequently diluted in 1 ml of 0.9% NaCl solution. The concentration of parathyroid hormone (PTH 1–84) in the aspirate was determined by electrochemiluminescence using the Elecsys analyser (cobas e 801). Absolute values of PTHw were compared to the most recent serum PTH concentration (PTHs) by calculating the PTHw-to-PTHs ratio. A positive result was defined as a PTHw-to-PTHs ratio ≥ 1.

All subjects also underwent radionuclide imaging consisting of two methods.

1. Planar subtraction scintigraphy using the Nucline gamma camera (Mediso, Hungary) was performed. The imaging protocol included the administration of two radiopharmaceuticals. Initially, 74 MBq of technetium-99m pertechnetate (^99m^Tc, generator: Polatom, Otwock, Poland) was injected to visualise tracer distribution within the thyroid gland. Planar images of the neck were acquired 15 minutes post-injection. This was followed by administration of 740 MBq of ^99m^Tc-MIBI (PoltechMIBI, Polatom, Otwock, Poland) using a dual-phase protocol, with anterior neck images obtained at 15 and 120 minutes. The thyroid scan was subtracted from the ^99m^Tc-MIBI images. Foci of parathyroid lesion were defined as areas with increased ^99m^Tc-MIBI uptake in the absence of increased ^99m^Tc-pertechnetate accumulation.
2. SPECT/CT images were registered subsequently, i.e. ca. 130 min after ^99m^Tc-MIBI injection using the Symbia Intevo Bold SPECT/CT scanner (Siemens) with acquisition time of 20 min.

The abbreviation “MIBI” in the manuscript refers to the total procedure including planar scan and SPECT/CT imaging.

To avoid any potential influence of needle aspiration on tracer distribution, PTHw sampling was invariably performed after MIBI imaging. To minimize operator bias, all PTHw procedures throughout the entire study period were performed by the same two highly experienced endocrinologists The results of the PTHw and MIBI were independently evaluated by three researchers. Findings were categorised as consistent, partially consistent, or inconsistent.

Statistical analyses were performed using Statistica 14 (Cloud Software Group, Inc. (2023)) and Microsoft Excel (2019). A p-value of <0.05 was considered statistically significant. Variables distribution was assessed with the Shapiro–Wilk test. Group comparisons were performed with the Mann–Whitney U test, with effect size estimated using the rank-biserial correlation coefficient (rg). We used Spearman’s rank correlation test and the Chi-square test with the maximum likelihood correction (Chi^2^ NW) to examine associations between variables. For the Chi^2^ NW test, effect size was determined using the Phi coefficient (φ) for 2×2 contingency tables (degrees of freedom [df] = 1), and Cramér’s V for tables with df > 1. Comparisons and associations between positive and negative PTHw results, as well as between negative and positive MIBI outcomes, were performed across the entire study cohort. In accordance with the requirements of the applied statistical tests, comparative and association analyses exploring the relationship between clinical and biochemical variables and imaging outcomes (PTHw and MIBI) were restricted to patients in whom a positive result was obtained with only one of the two modalities.

The study protocol was approved by the Bioethics Committee of the Poznań University of Medical Sciences, in accordance with applicable Polish regulations and with the Declaration of Helsinki.

## Results

### Group characteristics

Group characteristics are presented in Table 1. Notably, in 72 patients, the PTHw level was equal to or exceeded the upper limit of detection (2300 pg/mL).

**Table 1:** Group characteristics. data are presented as median and interquartile range (Median [Q1–Q3])

Parameter	Reference range	Whole group	PTHw (+), MIBI (-)	PTHw (-), MIBI (+)
PTH washout concentration (PTHw)	- (pg/mL)	2781 [526-5000]	3126 [282-5000]	19.80 [14.20-34]
PTHw-to-PTHs ratio	<1	18.66 [3.77-35.37]	16.15 [3.83-33.59]	0.18 [0.15-0.29]
serum PTH (PTHs)	15–57 (pg/mL)	121 [91.20-172]	121 [73.70-173]	91.70 [74-133.20]
total calcium (Ca)	8.80–10.20 (mg/dL)	11.17 [10.67-12.01]	11.19 [10.80-11.63]	10.92 [10.54-11.20]
ionized calcium (Ca2+)	4.20–5.20 (mg/dL)	6.11 [5.88-6.61]	6.10 [5.79-6.42]	5.92 [5.72-6.04]
inorganic phosphates (Pi)	2.70–4.50 (mg/dL)	2.72 [2.39-3.12]	2.91 [2.67-3.21]	2.76 [2.53-3.01]
vitamin D3 (25-OH-D)	30–80 (ng/mL)	27 [23-36]	27 [26-35]	33 [30-38]
Creatinine	0.50-0.90 (mg/dl)	0.79 [0.72-0.92]	0.77 [0.72-0.84]	0.84 [0.75-0.94]
24 h urinary Ca	100–320 (mg/24 h)	275.50 [183-430]	308.50 [208-427]	220 [118-233]
24 h urinary P	0.40–1.30 (g/24 h)	0.60 [0.50-0.80]	0.55 [0.45-0.70]	0.50 [0.40-0.60]
Alkaline phosphatase	35–105 (U/L)	86 [71-112]	105.50 [86-148]	84 [61-85]
Maximal lesion diameter in US [mm]	-	11.30 [8-15]	8.50 [7-10]	8 [6-13]

24 h urinary Ca -calcium in 24-h urine collection, 24 h urinary P -phosphates in 24-h urine collection, PTHs -serum PTH concentration.

### PTHw vs MIBI

PTHw yielded positive results in 106/125 (84.80%) patients. MIBI was considered positive in 105/125 (84.00%) patients.

A comprehensive analysis of the concordance between imaging modalities (PTHw and MIBI) revealed the following distribution: fully concordant – 78/125 (62.40%), partially concordant – 20/125 (16.00%), and discordant – 27/125 (21.60%). Due to the absence of surgical and histopathological confirmation in partially concordant cases, both imaging methods were considered positive for preoperative PL localization.

**Fig. 2:**
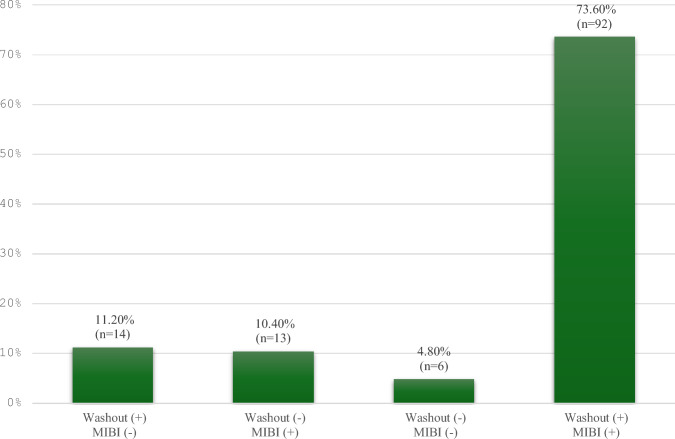
Comparison of preoperative imaging modalities for localization of parathyroid lesions causing primary hyperparathyroidism Washout - ultrasound-guided parathyroid hormone washout, MIBI - ^99m^Tc-MIBI+SPECT/CT

Figure 2 provides a comparative overview of the performance of preoperative imaging modalities in localizing PL. Within the group of patients with negative or inconclusive MIBI, PTHw enabled identification of the PL location in 14/20 (70.00%) cases. Conversely, among patients with negative PTHw results, 13/19 (68.42%) were MIBI-positive. In 92/106 (86.79%) patients with positive PTHw results, the PL was successfully visualised also in MIBI. In 92/105 (87.62%) MIBI -positive patients, preoperative localization was also indicated by PTHw.

### Localization of PTH-secreting parathyroid lesion

#### Most frequent localization

**Fig. 3:**
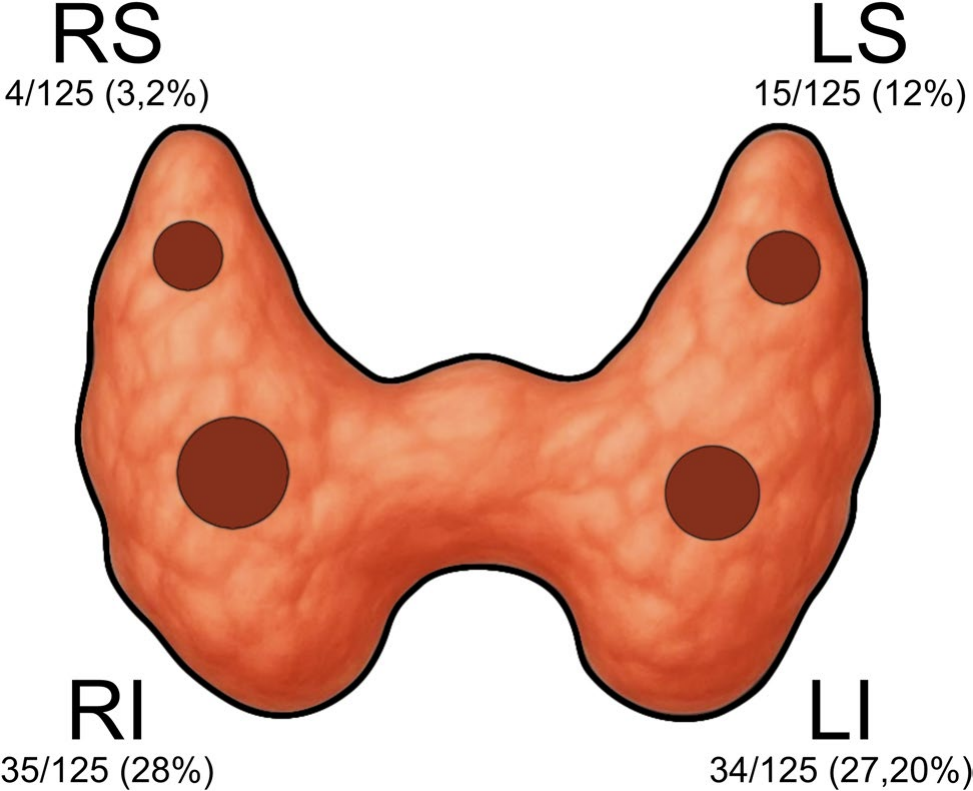
Frequency of parathyroid lesion responsible for primary hyperparathyroidism location using US-guided PTH washout (RS – right superior, RI – right inferior; LS – left superior; LI – left inferior)

The most frequently identified location of PL using ultrasound-guided PTHw was the right inferior gland, observed in 35 of 125 patients (28.00%). The frequency of lesions in other locations is presented in Fig. 3. Multiple lesions (12/125; 9.60%) and lesions of unspecified localization (25/125; 20.00%) are not depicted in this figure.

#### Multiple lesions

PTHw identified multiple lesions in 12 patients. In 4 of them, concordant findings were visualised by MIBI. Of the remaining eight cases, MIBI was positive in seven, indicating a solitary lesion.

MIBI identified multiple lesions in 9 patients. In 4 of these patients, ultrasound also suggested multiple lesions, and all were PTHw positive. The remaining 5/9 patients had PTHw positive for single lesion.

### Lesion size

**Fig. 4:**
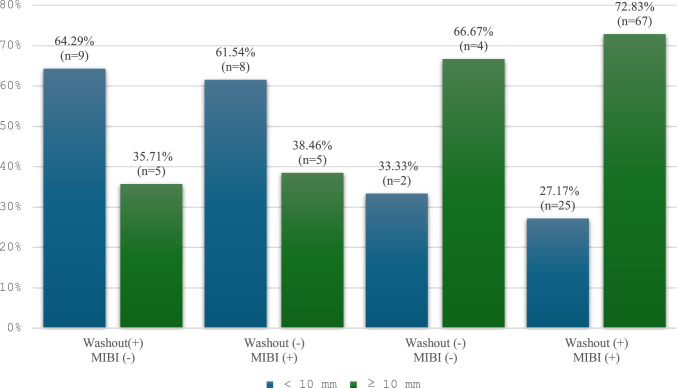
Comparison of PTH washout and ^99m^Tc-MIBI with SPECT/CT results according to the maximum diameter of PTH-secreting parathyroid lesion (determined by ultrasound) Washout - PTH washout, MIBI - ^99m^Tc-MIBI with SPECT/CT

To assess the impact of the maximal lesion diameter on imaging results, patients were divided into two groups: <10 mm in largest diameter (44/125; 35.20% ) and ≥ 10 mm (81/125; 64.80%) (Fig. 4). For lesions <10 mm, PTHw indicated PL localisation in 34/44 (77.27%), while MIBI in 33/44 (75.00%). In the ≥ 10 mm group, both modalities were positive in 67/81 (82.72%) cases.

**Fig. 5:**
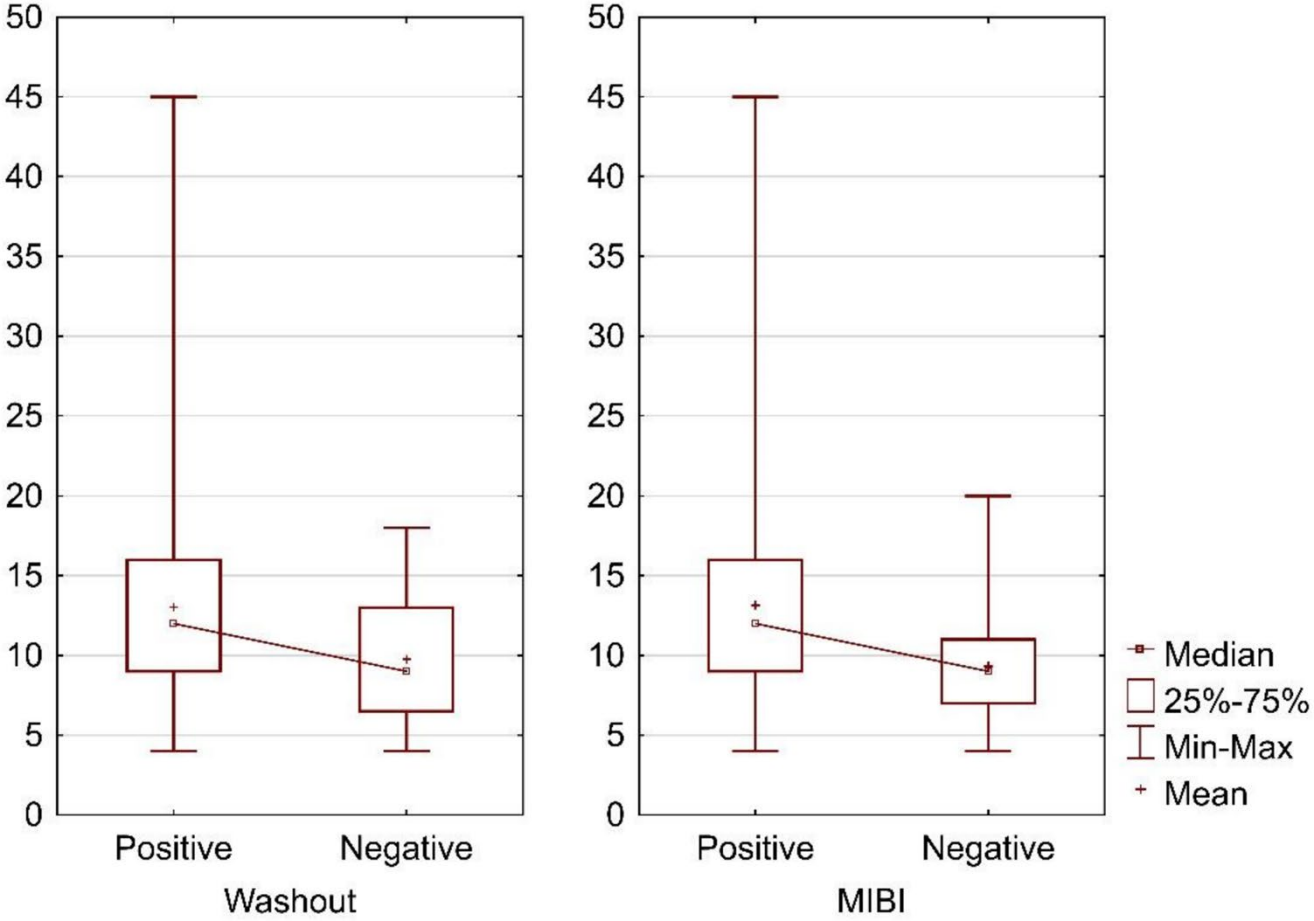
Comparison of the maximal lesion dimensions in patients with positive versus negative results of PTH washout (left) and 99mTc-MIBI SPECT/CT (right). Boxes represent the interquartile range (25th–75th percentile), horizontal lines indicate the median, whiskers denote the minimum and maximum values, and crosses indicate the mean Washout - PTH washout, MIBI - 99mTc-MIBI with SPECT/CT

Patients with positive results in either PTHw or MIBI had significantly larger lesion diameters compared to those with negative results. The median maximal lesion diameter for the PTHw-positive group was 12 mm [IQR 9–16], compared to 9 mm [IQR 6.50–13] in the PTHw-negative group. U = 692.50, p =0.03, r₍g₎ = 0.31, indicating a moderate effect size. For the MIBI -positive group, the median maximal lesion diameter was 12 mm [IQR 9–16], whereas in the MIBI -negative group it was 9 mm [IQR 7–11]. The effect size was also considered moderate, as U = 643, p =0.01, r₍g₎ = 0.39 (Fig. 5).

MIBI showed a statistically significant association with lesion size, with positive results more frequently observed when maximal PL diameter exceeded 10 mm (χ^2^ = 3.92, *p* = 0.048; φ = -0.18). Although the effect size was weak, this finding suggests that the lesion diameter may influence the sensitivity of MIBI in localizing lesion responsible for PHPT. This effect was not observed for PTHw.

### Concomitant thyroid and parathyroid disorders

**Fig. 6:**
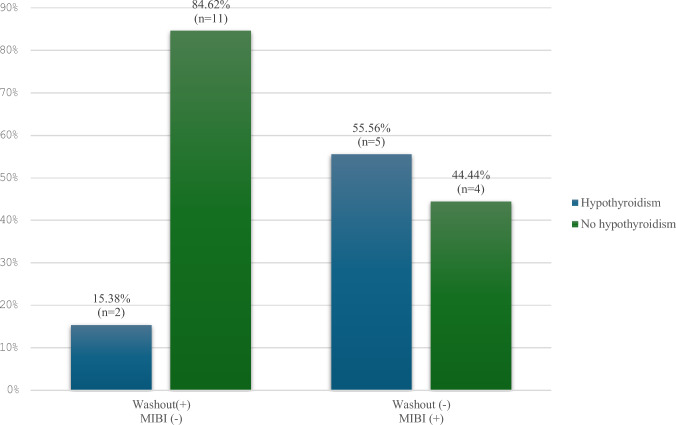
Discordant PTH washout and 99mTc-MIBI SPECT/CT results in relation to hypothyroidism Washout - PTH washout, MIBI - 99mTc-MIBI with SPECT/CT

Hypothyroidism was confirmed in 32 patients. Concordant results of PTHw and MIBI imaging were obtained in 25 individuals. Discordant findings between the two modalities are presented in Fig. 6. In a comparative analysis restricted to patients with a positive result by either modality, those in whom PL localization was established using MIBI were significantly more likely to present with hypothyroidism (χ^2^ = 3.99, p = 0.046). Despite the phi coefficient indicating a large effect size, further research is required due to the small sample size.

No significant associations were identified between multinodular goitre, autoimmune thyroiditis, prior thyroidectomy or parathyroidectomy and the method of preoperative PTH-secreting parathyroid lesion localization. These findings should be interpreted with caution and confirmed in studies involving larger patient populations.

### BMI

The median body mass index (BMI), based on 106 available results, was 26.62, with an interquartile range of 22.78–30.40. A total of 29 patients (27.36%) had a BMI greater than 30. Among them, 26/29 (89.66%) had a positive PTHw, while MIBI yielded positive results in 25/29 (86.21%). Statistical analysis did not reveal any significant differences or association between preoperative localization imaging method and BMI.

### Laboratory parameters analysis

#### PTHw measurement

We observed a significant positive correlation between PTH measurement in washout fluid and several parameters, including: serum PTH (r=0.29), PTHw-to-PTHs ratio (r=0.84), total calcium (r=0.27), ionized calcium (r=0.31), ALP (r=0.31) and maximal lesion diameter (r=0.25). Conversely, increasing PTHw values were associated with decreasing levels of phosphate (r = -0.22) and vitamin D (r = -0.20).

Patients with positive PTHw results had significantly higher PTHw levels (median 4474 [IQR 1862–5000]) compared to those with negative PTHw results (median 19.80 [IQR 9.08–34]); U=0, p<0.001, r(g)=1. Similarly, PTHw levels were significantly higher in patients with positive MIBI results (median 3267 [IQR 1155–5000]) versus MIBI-negative individuals (median 404 [IQR 72.90–4434]); U=702.50, p=0.02, r(g)=0.33. Patients with PL localization established via PTHw had higher PTHw levels (median 3126 [IQR 282–5000]) compared to those localized by MIBI (median 19.80 [IQR 14.20–34]); U=0, p<0.001, r(g)=1. The effect size was assessed as very strong for PTHw and moderate for MIBI.

#### Serum PTH

PTHs levels positively correlated with total calcium (r = 0.62), ionized calcium (r = 0.62), creatinine (r=0.22) and maximal lesion diameter (r = 0.52). An inverse correlation was observed between PTHs and both serum phosphate (r = -0.38) and vitamin D (r = -0.27).

Participants with positive PTHw presented significantly higher PTHs (median 123 [ IQR 92.20-174]) than those with negative PTHw (median 91.70 [IQR 70.40-135]); U = 666, p= 0.02, r₍g₎ = 0.34, indicating a moderate effect size.

#### PTHw-to-PTHs ratio

Obviously, significantly higher PTHw-to-PTHs ratio were present in patients with positive PTHw results (median 23.32 [IQR 9.94–38.17]) than in negative PTHw (median 0.20 [IQR 0.11–0.43]); U=0, p<0.001, r(g)=1. MIBI positive patients had a higher ratio (median 19.16 [IQR 5.20–36.83]) compared to those with negative MIBI (median 4.78 [IQR 0.58–26.83]); U=749.5, p=0.04, r(g)=0.29. Moreover, similar association in terms of PTHw-to-PTHs ratio was observed for PTHw-positive patients (median 16.15 [IQR 3.83–33.59]) in comparison to patients with PL localised by MIBI (median 0.18 [IQR 0.15–0.29]); U=0; p<0.001, r(g)=1. The effect size of this association was assessed as very strong for PTHw and moderate for MIBI.

#### Ionized calcium

Patients with positive PTHw results had significantly higher ionized calcium levels (median 6.15 [IQR 5.90–6.63]) than those with negative PTHw (median 5.94 [IQR 5.76–6.10]); U=554, p=0.02, r(g)=0.34.

#### Vitamin D3

Serum vitamin D3 levels were significantly lower in patients with positive PTHw results (median 27 [IQR 22–35]) compared to those with negative PTHw (median 33 [IQR 27.50–38.50]); U=543.50, p=0.04, r(g)=0.31.

### MEN1 Mutation Status

Among patients with a genetically confirmed MEN1 mutation (n = 9), all individuals (100%) demonstrated positive PTHw results, while MIBI was positive in 7 patients (77.78%). In the subgroup without MEN1 mutations (n = 116; MEN1-negative or not subjected to genetic testing), PL localization was established via PTHw in 97 patients (83.62%), whereas MIBI scintigraphy yielded positive results in 98 patients (84.48%). Concordant positive findings across both imaging modalities were observed in 85 patients without MEN1 mutation (73.28%).

## Discussion

### Comparison of US-guided PTHw to ^99m^Tc-MIBI with SPECT

Our findings indicate concordance and complementarity between PTHw and MIBI for preoperative localization of parathyroid lesions in PHPT.

PTHw is a safe, minimally invasive, and cost-effective modality, particularly valuable in centres lacking access to nuclear medicine facilities. It is especially advantageous for pregnant and lactating women, as well as for individuals with claustrophobia. FNAB remains a well-established diagnostic technique with a favourable safety profile and high diagnostic accuracy [26]. Although theoretical concerns regarding needle-tract tumour seeding have been raised, large analyses indicate that this complication is exceedingly rare (approximately 0.003-0.009%) and does not adversely affect prognosis [27–30]. The risk may depend on procedural factors, including biopsy site and the number of needle passes [31]. In parathyroid disease, PTHw is typically obtained through a single-needle puncture to confirm the PL. Given that parathyroid carcinoma accounts for less than 1% of cases, current evidence, including recent systematic reviews and meta-analyses by Castellana et al., supports the safety of this approach when performed with appropriate technique and clinical judgment [6, 25].

In contrast, MIBI offers high reproducibility without the need for biopsy, a feature of particular relevance in patients with obesity or ectopic parathyroid gland localization. In our study, localization rates were comparable for PTHw (84.8%) and MIBI (84.0%), with concordant findings in 73.6% of patients. Notably, PTHw localized the lesion in 70.0% of cases with negative or inconclusive MIBI, while MIBI was positive in 68.4% of patients with negative PTHw.

Our results are consistent with prior studies supporting the use of combined imaging modalities for preoperative localization in PHPT [18, 34–36]. The sensitivity and specificity of imaging techniques for PL evaluation vary widely across studies, ranging from 64–93% for US and 52.2–95% for MIBI [16–18, 20, 21, 24, 37–39]. A meta-analysis by Moghadam et al. demonstrated comparable sensitivity between ultrasound-based techniques and MIBI (80% vs. 83%), with no statistically significant difference.[37] Castellana et al. reported a pooled sensitivity of 95% for ultrasound-guided PTHw in PHPT patients with negative scintigraphy, a finding supported by subsequent studies [25, 35, 36]. The markedly lower sensitivity of MIBI versus PTHw reported by Obołończyk et al. (52.2% vs. 95.6%) may be attributable to the use of planar scintigraphy alone [24].

### Potential factors influencing diagnostic performance of US-guided PTHw or ^99m^Tc-MIBI SPECT/CT

The experiment provides a new insight into potential factors influencing diagnostic performance of US-guided PTHw and MIBI, in terms of specific clinical, biochemical, and anatomical variables.

#### Lesion size

Lesion size has been consistently recognized as one of key determinants of 99mTc-MIBI scintigraphy sensitivity [34]. Prior studies have demonstrated substantially higher localization accuracy for larger parathyroid tumours, particularly those exceeding 500 mg [17]. The incorporation of SPECT/CT into standard scintigraphic protocols has enhanced the detection of smaller lesions. However, lesion size and volume remained significant factors influencing localization success [40]. Consistent with these observations, we found that patients with positive results in either PTHw or MIBI presented with significantly larger lesions. MIBI positivity was significantly associated with lesion size, with higher detection rates for lesions exceeding 10 mm in maximal diameter, whereas no such association was observed for PTHw. This suggests that PTHw may be less size-dependent and therefore potentially more useful in the detection of smaller lesions, although these findings should be interpreted cautiously and viewed primarily as a trend, given the borderline statistical significance and modest effect size.

The size dependency of MIBI may be attributable to the relatively poor spatial resolution of the SPECT technique that is still higher than that of the planar images [41]. Parathyroid adenomas demonstrate ^99m^Tc-MIBI uptake due to increased number of active mitochondria that are responsible for the binding of the radiopharmaceutical in the cells [42]. Number of adenoma cells increases proportionately with the cube of the adenoma’s diameter increase. Therefore, larger lesions contain a much higher number of active mitochondria then smaller foci. It explains why smaller adenomas may exhibit insufficient tracer uptake or retention, increasing the risk of false-negative results. In contrast, PTHw offers size-independent diagnostic utility by directly measuring hormone concentration in aspirated fluid [43].

#### Concomitant thyroid disease

In our study, MIBI demonstrated superior localization performance among patients with concomitant hypothyroidism. However, multinodular goitre or autoimmune thyroiditis showed no significant association with preoperative imaging efficacy. These findings require cautious interpretation due to the limited size of the respective subgroups.

Evidence vary regarding the impact of coexisting thyroid disease on parathyroid imaging. Blanco-Saiz et al. observed no significant impact of thyroid pathology on PL detection by either MIBI or US [44]. In contrast, Barczyński et al. and Popowicz et al. reported reduced diagnostic accuracy of the scintigraphy and PTHw in the presence of nodular goitre or thyroiditis [45, 46]. Findings from Boi et al. and Erbil et al. supported the superior performance of ultrasound-guided PTHw in patients with coexisting thyroid disorders [47, 48]. These discrepancies likely reflect methodological variations, differences in imaging thresholds, and institutional expertise.

Potential explanation includes reduced ultrasound sensitivity in structurally altered thyroid tissue, which may impair accurate needle guidance for PTH washout, whereas scintigraphy is less dependent on anatomical definition and relies primarily on preserved mitochondrial tracer uptake [43].

#### Biochemical parameters

Biochemical profiles further supported the discriminative capacity of both techniques. Increasing PTHw values correlated positively with elevations in PTHs, PTHw-to-PTHs ratio, total and ionized calcium, and alkaline phosphatase (ALP). Conversely, PTHw values demonstrated an inverse correlation with serum phosphate and vitamin D3.

Patients with positive ultrasound-guided washout results demonstrated higher PTHs values, PTHw-to-PTHs ratios, and ionized calcium levels, alongside lower vitamin D3 levels. Those findings reinforce the physiological plausibility and diagnostic validity of the technique.

#### MEN1 mutation status

In our study, positive localization results with PTHw and MIBI among MEN1-negative or untested patients closely mirrored those observed in the overall cohort. The proportions of positive localization results remained consistent across analyses, with only minimal numerical variation. These findings suggest that MEN1-associated disease may not uniformly preclude the detection of PTH-secreting parathyroid lesion using standard preoperative localization techniques. This observation is clinically relevant in light of the multiglandular nature of MEN1-related primary hyperparathyroidism, which has traditionally been viewed as a major limitation of localization studies [49, 50]. Nevertheless, given the limited number of MEN1-positive patients and the lack of histopathological confirmation, these results should be interpreted cautiously and require validation in larger, genetically well-characterized cohorts.

### Radionuclide imaging

For many years, localization of PL has been performed using ^99m^Tc-MIBI scintigraphy. There are several variations of this method, all of them showing decent values of sensitivity and specificity [51]. Including SPECT/CT has increased these parameters even more [52]. For example, Woods et al. reported sensitivity and specificity for ^99m^Tc-MIBI SPECT/CT of 95% and 89%, respectively [53]. For several years, PL imaging using PET/CT with ^18^F-choline has become increasingly available. This technique showed even higher accuracy, although it is associated with higher radiation exposure and much higher cost [23, 54]. Therefore, ^18^F-choline PET/CT is usually employed in the case of negative scintigraphy [51]. In this study, it was not utilized due to limited availability during patient recruitment.

### Limitations

The retrospective design introduces potential selection bias, while the real-world nature of the study precluded blinding of clinicians performing the imaging assessments and may have introduced interpretation bias. On the other hand, applied methodology reflects better standard clinical procedure. PTHw washout procedure is operator-dependent, necessitating considerable experience in ultrasound imaging what may limit reproducibility of the results. It is worth noting that we aimed to assess the compatibility of PTHw and MIBI rather than to assess its diagnostic accuracy and therefore histopathological confirmation was not incorporated in the analysis. Notably, there is no standardized cut-off value for PTHw positivity, what limits interstudy comparability. This variability underscores the need for future prospective studies aimed at establishing validated criteria. In our cohort, a cut-off value of PTHw-to-PTHs ratio ≥1 was adopted, consistent with several previous studies [35, 55]. Korkmaz et al. reported 97.05% sensitivity, 94.29% PPV, and 91.67% diagnostic accuracy for this threshold [55].

## Conclusions

In conclusion, our findings reinforce the complementary role of ultrasound-guided PTH washout and 99mTc-MIBI scintigraphy with SPECT/CT in the preoperative PHPT diagnostic evaluation. We observed a trend suggesting an association between lesion size and imaging results for both MIBI and PTHw. However, a statistically significant association with lesion size was observed only for MIBI, with positive results more frequently observed when maximal lesion diameter exceeded 10 mm. Our results highlight the importance of selecting imaging strategies based on individual patient profiles to improve preoperative localization. These findings should be interpreted with caution and further research involving larger, histopathologically verified cohorts is needed to strengthen these observations and guide clinical decision-making.

## Data Availability

The datasets generated and/or analyzed during the current study are not publicly available due to confidentiality concerns, but they can be accessed from the corresponding author upon reasonable request.
